# Diethyl 4,6-diacetamido­isophthalate

**DOI:** 10.1107/S1600536811013845

**Published:** 2011-04-22

**Authors:** Peishen Li, Xianghui Li, Chao Chen, Lihua Yuan, Wen Feng

**Affiliations:** a Key Laboratory for Radiation Physics and Technology of the Ministry of Education, College of Chemistry, Institute of Nuclear Science and Technology, Sichuan University, Chengdu 610064, Sichuan, People’s Republic of China

## Abstract

In the title compound, C_16_H_20_N_2_O_6_, two intra­molecular N—H⋯O hydrogen bonds occur, in which the carbonyl O atoms of the ethyl acetate groups serve as the acceptor atoms; both motifs generate *S*(6) rings. In the crystal, mol­ecules are linked by weak C—H⋯O links (with the acceptor O atoms part of the amide groups), generating [001] chains.

## Related literature

For background to intra­molecular hydrogen bonds this class of compound, see: Zhu *et al.* (2000 ▶); Yuan *et al.* (2004 ▶); Feng *et al.* (2009 ▶); Yan *et al.* (2010 ▶); Zhang *et al.* (2008 ▶). For a related structure, see: Zhang *et al.* (2006 ▶).
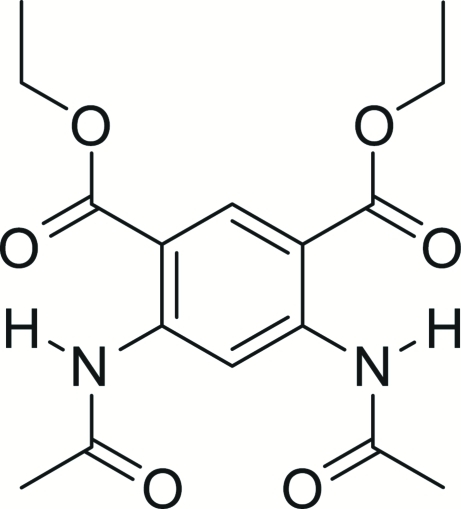


## Experimental

### 

#### Crystal data

C_16_H_20_N_2_O_6_
*M* *_r_* = 336.34
Triclinic, 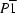

*a* = 7.951 (3) Å
*b* = 10.249 (3) Å
*c* = 11.109 (4) Å
α = 76.70 (3)°
β = 77.42 (3)°
γ = 76.50 (2)°
*V* = 843.7 (5) Å^3^
*Z* = 2
Mo *K*α radiation
μ = 0.10 mm^−1^
*T* = 292 K
0.50 × 0.46 × 0.40 mm

#### Data collection

Enraf–Nonius CAD-4 diffractometer
3149 measured reflections
3094 independent reflections
1826 reflections with *I* > 2σ(*I*)
*R*_int_ = 0.004
3 standard reflections every 150 reflections  intensity decay: 4.5%

#### Refinement

*R*[*F* ^2^ > 2σ(*F* ^2^)] = 0.079
*wR*(*F* ^2^) = 0.268
*S* = 1.09
3094 reflections
223 parameters
4 restraints
H atoms treated by a mixture of independent and constrained refinement
Δρ_max_ = 0.59 e Å^−3^
Δρ_min_ = −0.65 e Å^−3^

### 

Data collection: *DIFRAC* (Gabe *et al.*, 1993 ▶); cell refinement: *DIFRAC*; data reduction: *NRCVAX* (Gabe *et al.*, 1989 ▶); program(s) used to solve structure: *SHELXS97* (Sheldrick, 2008 ▶); program(s) used to refine structure: *SHELXL97* (Sheldrick, 2008 ▶); molecular graphics: *ORTEP-3* (Farrugia, 1997 ▶); software used to prepare material for publication: *SHELXL97*.

## Supplementary Material

Crystal structure: contains datablocks global, I. DOI: 10.1107/S1600536811013845/hb5828sup1.cif

Structure factors: contains datablocks I. DOI: 10.1107/S1600536811013845/hb5828Isup2.hkl

Additional supplementary materials:  crystallographic information; 3D view; checkCIF report

## Figures and Tables

**Table 1:** Hydrogen-bond geometry (Å, °)

*D*—H⋯*A*	*D*—H	H⋯*A*	*D*⋯*A*	*D*—H⋯*A*
N1—H1*N*⋯O2	0.88 (4)	1.92 (3)	2.676 (4)	144 (3)
N2—H2*N*⋯O6	0.81 (4)	1.96 (4)	2.656 (4)	143 (3)
C9—H9*B*⋯O3^i^	0.96	2.54	3.445 (6)	157
C16—H16*A*⋯O4^ii^	0.96	2.57	3.485 (7)	160

Symmetry codes: (i) 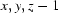; (ii) 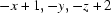.

**Fig. 1.**
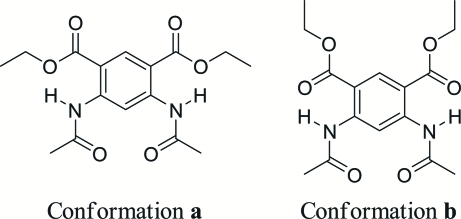
The two possible intramolecular hydrogen bond conformations of the title compound.

**Fig. 2.**
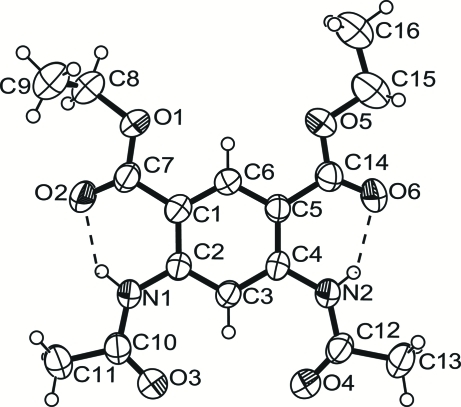
A view of the title compound with displacement ellipsoids shown at the 50% probability level. Hydrogen bonds are indicated by broken lines.

## supplementary crystallographic information

### Comment

Hydrogen bonds play a vital role in assisting formation of aromatic oligoamide foldmers and macrocycles (Zhu *et al.*, 2000; Yuan *et al.*, 2004; Feng *et al.*, 2009). It is important to examine the structural feature of the backbones that is responsible for the formation of foldmers and macrocycles. Research has focused on the role of intramolecular hydrogen bonds in regulating conformations and interactions both in solution and the solid state (Yan *et al.*, 2010; Zhang *et al.* 2008). Herein, we report on the crystal structure of the aromatic monomer containing intramolecular hydrogen bonds, which could be applied as important building blocks to construct macrocycles.

The title compound comprises two similar six-member hydrogen bonds. There are two possible comformations: (*a*) one with the ethoxy O atoms involving in the intramolecular NH···OC_2_H_5_ six-member hydrogen bonds (conformation a), and (*b*) the other with carbonyl O atoms that form intramolecualr H-bonds (conformation b) (Fig. 1). However, the crystal structure revealed the existence of two six-member hydrogen bonds that involve carbonyl O atoms, indicating that conformation b is more stable and is sustained by two intromolecular hydrogen bond N1H1···O2 (Table1, Fig. 2). It is expected that this type of hydrogen bonds may be exploited to construct macrocyles and supamolecular architecture with folded conformations. In the crystal, the molecules are linked by C—H···O interactions.

### Experimental

For synthesis of the title compound, Pd/C (50 mg) was added to the solution of diethyl 4,6-dinitroisophthalate (500 mg, 1.60 mmol) in CH_2_Cl_2_ and the mixture was stirred at room temperature under H_2_ atmosphere for 6 h. After removal of Pd/C and solvent, the white solid was obtained and then dissolved in CH_2_Cl_2_ (50 ml). Et_3_N (335 mg, 3.31 mmol) was added to the above solution followed by dropping acetyl chloride (350 mg, 4.46 mmol). After stirring 2 h at room temperature, the mixture was washed with distilled water, dried over anhydrous Na_2_SO_4_. Removal of solvent under reduced pressure gave the crude product, which was recrystallized from methanol to yield the white product (430 mg, yield 79.8%). ^1^H NMR (400 MHz, CDCl_3_): δ 10.95 (s, 2H), 9.98 (s, 1H), 8.74 (s, 1H), 4.35 (m, 4H), 2.25 (s, 6H), 1.20 (t, J = 6.4 Hz, 6H).

The crystal of the title compound was grown from the THF solution. This compound was dissolved in THF and filtered, then the filtrate was allowed to stand undisturbed to give the single-crystal.

### Refinement

The H atoms bonded to atom N1 and N2 of the title compound were located from difference Fourier maps and refined isotropically [N1—H = 1.88 (4), N2—H = 0.81 (4) Å]. H atoms bonded to C atoms were placed in calculated positions and refined using a riding model, with C—H = 0.9300 Å and *U*_iso_(H) = 1.2Ueq(C) for aryl H atoms, C—H = 0.9700 Å and *U*_iso_(H) = 1.2Ueq(C) for methylene H atoms, C—H = 0.9600 Å and *U*_iso_(H) = 1.5Ueq(C) for methyl H atoms. The deepest difference hole of -0.65 e.Å^-3^ is 0.33 Å from atom C16.

### Crystal data

**Table:**

C_16_H_20_N_2_O_6_	*Z* = 2
*M**_r_* = 336.34	*F*(000) = 356
Triclinic, *P*1	*D*_x_ = 1.324 Mg m^−^^3^
*a* = 7.951 (3) Å	Mo *K*α radiation, λ = 0.71073 Å
*b* = 10.249 (3) Å	Cell parameters from 20 reflections
*c* = 11.109 (4) Å	θ = 5.4–6.7°
α = 76.70 (3)°	µ = 0.10 mm^−^^1^
β = 77.42 (3)°	*T* = 292 K
γ = 76.50 (2)°	Block, colourless
*V* = 843.7 (5) Å^3^	0.50 × 0.46 × 0.40 mm

### Data collection

**Table:**

Enraf–Nonius CAD-4 diffractometer	*R*_int_ = 0.004
Radiation source: fine-focus sealed tube	θ_max_ = 25.5°, θ_min_ = 1.9°
graphite	*h* = −9→9
ω/2θ scans	*k* = −1→12
3149 measured reflections	*l* = −12→13
3094 independent reflections	3 standard reflections every 150 reflections
1826 reflections with *I* > 2σ(*I*)	intensity decay: 4.5%

### Refinement

**Table:**

Refinement on *F*^2^	Primary atom site location: structure-invariant direct methods
Least-squares matrix: full	Secondary atom site location: difference Fourier map
*R*[*F*^2^ > 2σ(*F*^2^)] = 0.079	Hydrogen site location: inferred from neighbouring sites
*wR*(*F*^2^) = 0.268	H atoms treated by a mixture of independent and constrained refinement
*S* = 1.09	*w* = 1/[σ^2^(*F*_o_^2^) + (0.1639*P*)^2^ + 0.1078*P*] where *P* = (*F*_o_^2^ + 2*F*_c_^2^)/3
3094 reflections	(Δ/σ)_max_ < 0.001
223 parameters	Δρ_max_ = 0.59 e Å^−^^3^
4 restraints	Δρ_min_ = −0.65 e Å^−^^3^

### Special details

**Table:**

Geometry. All e.s.d.'s (except the e.s.d. in the dihedral angle between two l.s. planes) are estimated using the full covariance matrix. The cell e.s.d.'s are taken into account individually in the estimation of e.s.d.'s in distances, angles and torsion angles; correlations between e.s.d.'s in cell parameters are only used when they are defined by crystal symmetry. An approximate (isotropic) treatment of cell e.s.d.'s is used for estimating e.s.d.'s involving l.s. planes.
Refinement. Refinement of *F*^2^ against ALL reflections. The weighted *R*-factor *wR* and goodness of fit *S* are based on *F*^2^, conventional *R*-factors *R* are based on *F*, with *F* set to zero for negative *F*^2^. The threshold expression of *F*^2^ > σ(*F*^2^) is used only for calculating *R*-factors(gt) *etc*. and is not relevant to the choice of reflections for refinement. *R*-factors based on *F*^2^ are statistically about twice as large as those based on *F*, and *R*- factors based on ALL data will be even larger.

### Fractional atomic coordinates and isotropic or equivalent isotropic displacement parameters (Å^2^)

**Table:**

	*x*	*y*	*z*	*U*_iso_*/*U*_eq_	
O1	0.9467 (4)	−0.1465 (3)	0.6464 (2)	0.0692 (9)	
O2	1.1334 (4)	−0.2976 (3)	0.7586 (2)	0.0706 (9)	
O3	1.0473 (5)	−0.3487 (3)	1.2179 (3)	0.0988 (12)	
O4	0.7415 (4)	−0.0447 (3)	1.3243 (3)	0.0796 (9)	
O5	0.5572 (4)	0.2164 (3)	0.7774 (3)	0.0692 (8)	
O6	0.5009 (4)	0.2628 (3)	0.9676 (3)	0.0718 (8)	
N1	1.0637 (4)	−0.3063 (3)	1.0068 (3)	0.0503 (8)	
H1N	1.113 (4)	−0.338 (4)	0.938 (3)	0.046 (9)*	
N2	0.6413 (4)	0.0776 (3)	1.1468 (3)	0.0514 (8)	
H2N	0.581 (5)	0.150 (4)	1.120 (3)	0.044 (10)*	
C1	0.9150 (4)	−0.1332 (4)	0.8583 (3)	0.0477 (8)	
C2	0.9442 (4)	−0.1847 (3)	0.9824 (3)	0.0427 (8)	
C3	0.8541 (4)	−0.1138 (3)	1.0777 (3)	0.0440 (8)	
H3	0.8745	−0.1477	1.1594	0.053*	
C4	0.7336 (4)	0.0075 (3)	1.0518 (3)	0.0413 (8)	
C5	0.7021 (4)	0.0591 (3)	0.9291 (3)	0.0449 (8)	
C6	0.7941 (4)	−0.0127 (3)	0.8356 (3)	0.0460 (8)	
H6	0.7736	0.0216	0.7539	0.055*	
C7	1.0103 (5)	−0.2022 (4)	0.7527 (3)	0.0539 (9)	
C8	1.0398 (7)	−0.1987 (5)	0.5319 (4)	0.0871 (15)	
H8A	1.1655	−0.2192	0.5310	0.104*	
H8B	1.0160	−0.1303	0.4581	0.104*	
C9	0.9772 (8)	−0.3269 (6)	0.5304 (5)	0.1008 (17)	
H9A	1.0175	−0.3987	0.5961	0.151*	
H9B	1.0233	−0.3543	0.4507	0.151*	
H9C	0.8512	−0.3090	0.5434	0.151*	
C10	1.1082 (5)	−0.3819 (4)	1.1169 (4)	0.0574 (10)	
C11	1.2385 (6)	−0.5100 (4)	1.1039 (4)	0.0680 (11)	
H11A	1.2382	−0.5696	1.1844	0.102*	
H11B	1.3533	−0.4888	1.0722	0.102*	
H11C	1.2081	−0.5544	1.0466	0.102*	
C12	0.6424 (5)	0.0486 (4)	1.2735 (3)	0.0544 (9)	
C13	0.5091 (6)	0.1480 (5)	1.3433 (4)	0.0723 (12)	
H13A	0.3951	0.1530	1.3249	0.108*	
H13B	0.5399	0.2367	1.3177	0.108*	
H13C	0.5073	0.1177	1.4320	0.108*	
C14	0.5778 (5)	0.1888 (4)	0.8965 (4)	0.0531 (9)	
C15	0.4366 (9)	0.3443 (6)	0.7425 (5)	0.1109 (14)	
H15A	0.4848	0.4216	0.7463	0.133*	
H15B	0.3245	0.3465	0.7987	0.133*	
C16	0.4146 (9)	0.3488 (6)	0.6092 (5)	0.1109 (14)	
H16A	0.3796	0.2666	0.6051	0.166*	
H16B	0.5239	0.3565	0.5534	0.166*	
H16C	0.3262	0.4263	0.5848	0.166*	

### Atomic displacement parameters (Å^2^)

**Table:**

	*U*^11^	*U*^22^	*U*^33^	*U*^12^	*U*^13^	*U*^23^
O1	0.0823 (19)	0.0772 (19)	0.0456 (15)	0.0082 (15)	−0.0164 (13)	−0.0255 (13)
O2	0.0655 (17)	0.0820 (19)	0.0598 (17)	0.0178 (15)	−0.0127 (13)	−0.0357 (14)
O3	0.130 (3)	0.084 (2)	0.0577 (19)	0.040 (2)	−0.0244 (18)	−0.0185 (16)
O4	0.094 (2)	0.082 (2)	0.0529 (16)	0.0193 (18)	−0.0208 (15)	−0.0222 (14)
O5	0.0774 (19)	0.0613 (16)	0.0625 (17)	0.0094 (14)	−0.0272 (14)	−0.0076 (13)
O6	0.083 (2)	0.0520 (15)	0.0749 (19)	0.0166 (14)	−0.0225 (15)	−0.0227 (14)
N1	0.0532 (17)	0.0460 (16)	0.0500 (17)	0.0046 (13)	−0.0107 (14)	−0.0184 (13)
N2	0.0526 (18)	0.0501 (18)	0.0507 (18)	0.0036 (15)	−0.0115 (14)	−0.0189 (14)
C1	0.0476 (19)	0.0513 (19)	0.0465 (19)	−0.0098 (16)	−0.0054 (15)	−0.0161 (15)
C2	0.0392 (17)	0.0438 (17)	0.0469 (18)	−0.0060 (14)	−0.0068 (14)	−0.0147 (14)
C3	0.0455 (18)	0.0449 (18)	0.0428 (18)	−0.0052 (15)	−0.0074 (14)	−0.0147 (14)
C4	0.0353 (16)	0.0430 (17)	0.0480 (18)	−0.0066 (14)	−0.0029 (13)	−0.0182 (14)
C5	0.0436 (18)	0.0433 (18)	0.0492 (19)	−0.0035 (15)	−0.0102 (15)	−0.0143 (15)
C6	0.0485 (19)	0.0480 (18)	0.0430 (17)	−0.0067 (15)	−0.0100 (14)	−0.0120 (14)
C7	0.055 (2)	0.058 (2)	0.051 (2)	−0.0040 (18)	−0.0062 (16)	−0.0240 (17)
C8	0.105 (4)	0.096 (3)	0.054 (2)	0.007 (3)	−0.009 (2)	−0.032 (2)
C9	0.121 (4)	0.107 (4)	0.081 (3)	0.002 (3)	−0.026 (3)	−0.048 (3)
C10	0.058 (2)	0.051 (2)	0.062 (2)	0.0021 (17)	−0.0145 (18)	−0.0186 (18)
C11	0.076 (3)	0.048 (2)	0.077 (3)	0.0043 (19)	−0.021 (2)	−0.0148 (19)
C12	0.056 (2)	0.060 (2)	0.050 (2)	−0.0067 (18)	−0.0058 (17)	−0.0237 (17)
C13	0.071 (3)	0.081 (3)	0.063 (2)	0.005 (2)	−0.005 (2)	−0.035 (2)
C14	0.058 (2)	0.0453 (19)	0.058 (2)	−0.0045 (17)	−0.0150 (17)	−0.0146 (16)
C15	0.130 (3)	0.087 (3)	0.103 (3)	0.021 (2)	−0.050 (3)	−0.008 (2)
C16	0.130 (3)	0.087 (3)	0.103 (3)	0.021 (2)	−0.050 (3)	−0.008 (2)

### Geometric parameters (Å, °)

**Table:**

O1—C7	1.338 (4)	C5—C14	1.482 (5)
O1—C8	1.474 (5)	C6—H6	0.9300
O2—C7	1.212 (4)	C8—C9	1.514 (6)
O3—C10	1.212 (5)	C8—H8A	0.9700
O4—C12	1.204 (5)	C8—H8B	0.9700
O5—C14	1.326 (4)	C9—H9A	0.9600
O5—C15	1.459 (5)	C9—H9B	0.9600
O6—C14	1.192 (4)	C9—H9C	0.9600
N1—C10	1.359 (5)	C10—C11	1.485 (5)
N1—C2	1.390 (4)	C11—H11A	0.9600
N1—H1N	0.88 (4)	C11—H11B	0.9600
N2—C12	1.371 (5)	C11—H11C	0.9600
N2—C4	1.391 (4)	C12—C13	1.505 (5)
N2—H2N	0.81 (4)	C13—H13A	0.9600
C1—C6	1.386 (5)	C13—H13B	0.9600
C1—C2	1.408 (5)	C13—H13C	0.9600
C1—C7	1.482 (5)	C15—C16	1.517 (7)
C2—C3	1.393 (4)	C15—H15A	0.9700
C3—C4	1.395 (4)	C15—H15B	0.9700
C3—H3	0.9300	C16—H16A	0.9600
C4—C5	1.398 (5)	C16—H16B	0.9600
C5—C6	1.388 (4)	C16—H16C	0.9600
			
C7—O1—C8	117.5 (3)	H9A—C9—H9B	109.5
C14—O5—C15	114.2 (3)	C8—C9—H9C	109.5
C10—N1—C2	130.8 (3)	H9A—C9—H9C	109.5
C10—N1—H1N	117 (2)	H9B—C9—H9C	109.5
C2—N1—H1N	112 (2)	O3—C10—N1	123.3 (3)
C12—N2—C4	131.2 (3)	O3—C10—C11	122.3 (4)
C12—N2—H2N	116 (3)	N1—C10—C11	114.4 (3)
C4—N2—H2N	112 (3)	C10—C11—H11A	109.5
C6—C1—C2	118.0 (3)	C10—C11—H11B	109.5
C6—C1—C7	119.7 (3)	H11A—C11—H11B	109.5
C2—C1—C7	122.3 (3)	C10—C11—H11C	109.5
N1—C2—C3	121.5 (3)	H11A—C11—H11C	109.5
N1—C2—C1	118.7 (3)	H11B—C11—H11C	109.5
C3—C2—C1	119.9 (3)	O4—C12—N2	124.0 (3)
C2—C3—C4	120.7 (3)	O4—C12—C13	122.9 (4)
C2—C3—H3	119.6	N2—C12—C13	113.1 (3)
C4—C3—H3	119.6	C12—C13—H13A	109.5
N2—C4—C3	121.0 (3)	C12—C13—H13B	109.5
N2—C4—C5	119.0 (3)	H13A—C13—H13B	109.5
C3—C4—C5	120.0 (3)	C12—C13—H13C	109.5
C6—C5—C4	118.3 (3)	H13A—C13—H13C	109.5
C6—C5—C14	119.7 (3)	H13B—C13—H13C	109.5
C4—C5—C14	122.0 (3)	O6—C14—O5	121.7 (3)
C1—C6—C5	123.0 (3)	O6—C14—C5	125.2 (3)
C1—C6—H6	118.5	O5—C14—C5	113.1 (3)
C5—C6—H6	118.5	O5—C15—C16	106.1 (4)
O2—C7—O1	122.4 (3)	O5—C15—H15A	110.5
O2—C7—C1	125.2 (3)	C16—C15—H15A	110.5
O1—C7—C1	112.4 (3)	O5—C15—H15B	110.5
O1—C8—C9	108.6 (4)	C16—C15—H15B	110.5
O1—C8—H8A	110.0	H15A—C15—H15B	108.7
C9—C8—H8A	110.0	C15—C16—H16A	109.5
O1—C8—H8B	110.0	C15—C16—H16B	109.5
C9—C8—H8B	110.0	H16A—C16—H16B	109.5
H8A—C8—H8B	108.3	C15—C16—H16C	109.5
C8—C9—H9A	109.5	H16A—C16—H16C	109.5
C8—C9—H9B	109.5	H16B—C16—H16C	109.5
			
C10—N1—C2—C3	4.6 (6)	C14—C5—C6—C1	178.7 (3)
C10—N1—C2—C1	−175.5 (3)	C8—O1—C7—O2	−4.3 (6)
C6—C1—C2—N1	179.3 (3)	C8—O1—C7—C1	175.0 (3)
C7—C1—C2—N1	−1.5 (5)	C6—C1—C7—O2	170.3 (4)
C6—C1—C2—C3	−0.8 (5)	C2—C1—C7—O2	−8.9 (6)
C7—C1—C2—C3	178.4 (3)	C6—C1—C7—O1	−9.1 (5)
N1—C2—C3—C4	−179.5 (3)	C2—C1—C7—O1	171.8 (3)
C1—C2—C3—C4	0.6 (5)	C7—O1—C8—C9	83.1 (5)
C12—N2—C4—C3	−1.0 (6)	C2—N1—C10—O3	−2.1 (7)
C12—N2—C4—C5	178.0 (3)	C2—N1—C10—C11	177.7 (3)
C2—C3—C4—N2	179.0 (3)	C4—N2—C12—O4	6.1 (7)
C2—C3—C4—C5	0.0 (5)	C4—N2—C12—C13	−175.2 (3)
N2—C4—C5—C6	−179.4 (3)	C15—O5—C14—O6	1.0 (6)
C3—C4—C5—C6	−0.4 (5)	C15—O5—C14—C5	−179.4 (4)
N2—C4—C5—C14	2.1 (5)	C6—C5—C14—O6	−174.9 (4)
C3—C4—C5—C14	−178.9 (3)	C4—C5—C14—O6	3.6 (6)
C2—C1—C6—C5	0.4 (5)	C6—C5—C14—O5	5.6 (5)
C7—C1—C6—C5	−178.8 (3)	C4—C5—C14—O5	−176.0 (3)
C4—C5—C6—C1	0.2 (5)	C14—O5—C15—C16	−173.5 (4)

### Hydrogen-bond geometry (Å, °)

**Table:**

*D*—H···*A*	*D*—H	H···*A*	*D*···*A*	*D*—H···*A*
N1—H1N···O2	0.88 (4)	1.92 (3)	2.676 (4)	144 (3)
N2—H2N···O6	0.81 (4)	1.96 (4)	2.656 (4)	143 (3)
C9—H9B···O3^i^	0.96	2.54	3.445 (6)	157
C16—H16A···O4^ii^	0.96	2.57	3.485 (7)	160

Symmetry codes: (i) *x*, *y*, *z*−1; (ii) −*x*+1, −*y*, −*z*+2.

## References

[bb1] Farrugia, L. J. (1997). *J. Appl. Cryst.* **30**, 565.

[bb2] Feng, W., Yamato, K., Yang, L. Q., Ferguson, J. S., Zhong, L. J., Zou, S. L., Yuan, L. H., Zeng, X. C. & Gong, B. (2009). *J. Am. Chem. Soc.* **131**, 2629–2637. 10.1021/ja807935y 19191583

[bb3] Gabe, E. J., Le Page, Y., Charland, J.-P., Lee, F. L. & White, P. S. (1989). *J. Appl. Cryst.* **22**, 384–387.

[bb4] Gabe, E. J., White, P. S. & Enright, G. D. (1993). *DIFRAC* American Crystallographic Association, Pittsburgh Meeting Abstract, PA 104.

[bb5] Sheldrick, G. M. (2008). *Acta Cryst.* A**64**, 112–122. 10.1107/S0108767307043930 18156677

[bb6] Yan, Y., Qin, B., Ren, C. L., Chen, X. Y., Yip, Y. K., Ye, R. J., Zhang, D. W., Su, H. B. & Zeng, H. Q. (2010). *J. Am. Chem. Soc.* **132**, 5869–5879. 10.1021/ja100579z 20364840

[bb7] Yuan, L. H., Feng, W., Yamato, K., Sanford, A. R., Xu, D. G., Guo, H. & Gong, B. (2004). *J. Am. Chem. Soc.* **126**, 11120–11121. 10.1021/ja0474547 15355071

[bb8] Zhang, A. M., Han, Y. H., Yamato, K., Zeng, X. C. & Gong, B. (2006). *Org. Lett.* **8**, 803–806. 10.1021/ol0526322 16494445

[bb9] Zhang, Y. F., Yamato, K., Zhong, K., Zhu, J., Deng, J. G. & Gong, B. (2008). *Org. Lett.* **10**, 4338–4342. 10.1021/ol801410f 18783228

[bb10] Zhu, J., Parra, R. D., Zeng, H. Q., Skrzypczak-Jankun, E., Zeng, X. C. & Gong, B. (2000). *J. Am. Chem. Soc.* **122**, 4219–4220.

